# Intrinsic and induced metabolic signatures underpin aluminum tolerance in bread wheat: a comparative metabolomics approach

**DOI:** 10.1007/s12298-025-01622-1

**Published:** 2025-07-22

**Authors:** Şükrü Serter Çatav, Emine Sonay Elgin, Köksal Küçükakyüz, Çağdaş Dağ

**Affiliations:** 1 Department of Biology, Faculty of Science, Muğla Sıtkı Koçman University, Muğla, Turkey; 2 Department of Chemistry, Faculty of Science, Muğla Sıtkı Koçman University, Muğla, Turkey; 3 Nanofabrication and Nanocharacterization Centre for Scientific and Technological Advanced Research, Koç University, Istanbul, Turkey; 4 Koç University İşBank Centre for Infectious Diseases, Koç University, Istanbul, Turkey

**Keywords:** Aluminum toxicity, Metabolomics, NMR spectroscopy, Root growth, Tolerance, Wheat

## Abstract

Aluminum (Al) toxicity is a major impediment to plant growth and yield in low pH soils. Exclusion and/or vacuolar sequestration of Al with organic acids and phenolic compounds is the primary tolerance mechanism utilized by plants to mitigate Al toxicity. However, little is known about the intrinsic and Al-induced metabolic differences underlying intraspecific variability in tolerance to Al toxicity. To fill this gap, we determined root metabolic profiles of Al-sensitive (Golia-99) and Al-tolerant (Demir-2000) bread wheat cultivars treated with 0, 10, and 30 µM AlCl_3_·6H_2_O using nuclear magnetic resonance (NMR) spectroscopy. Our results showed that there were marked differences in the concentrations of numerous metabolites between Golia-99 and Demir-2000 roots under both control and Al stress conditions. In this regard, a number of metabolites from the amino acid and TCA groups, such as citrate, cysteine, glutamate, isocitrate, phenylalanine, and succinate, were found to be intrinsically higher levels in Demir-2000 than in Golia-99. In addition, Al toxicity led to the accumulation of asparagine, glutamine, putrescine, pyroglutamate, and soluble sugars in Demir-2000 roots. Furthermore, Al treatments significantly altered many metabolic pathways in both cultivar-specific and cultivar-independent manners. The major pathways contributing to the difference in Al toxicity tolerance between Demir-2000 and Golia-99 were arginine biosynthesis, glycolysis/gluconeogenesis, and the metabolisms of cysteine and methionine, glutathione, glycine, serine and threonine, pyruvate, sulfur, and tyrosine. Overall, our results suggest that the distinct patterns of Al-induced overrepresentation in amino acid, carbohydrate, and energy metabolism play an important role in explaining the differential tolerance capacities of Demir-2000 and Golia-99 to Al toxicity. The outcomes of this study may provide valuable insights into improving Al tolerance in wheat through breeding and genetic engineering.

**Supplementary Information:**

The online version contains supplementary material available at 10.1007/s12298-025-01622-1.

## Introduction

Aluminum (Al) is one of the most abundant and ubiquitous elements in the crust of the Earth. The monomeric form of Al (Al(H_2_O)_6_^3+^ or Al^3+^) is solubilized in the soil under acidic conditions (pH < 5), resulting in phytotoxic effects (Kochian et al. 2015; Zhou et al. 2024). The earliest symptom of Al toxicity is the inhibition of root growth, which occurs within minutes at micromolar concentrations (Luo et al. 2024). Excess Al has been shown to initially suppress root cell expansion and elongation, and subsequently cell division (Kochian et al. 2004). In addition, Al^3+^ ions are known to bind with high affinity to the negatively charged free carboxyl groups of pectin, thereby affecting the structural and mechanical properties of the cell wall (Jaskowiak et al. 2019). Furthermore, the deleterious effects of Al toxicity on plant roots encompass alterations in membrane fluidity, impairment of the trans-membrane H^+^ gradient, inhibition of cation uptake, promotion of callose synthesis, disruption of signal transduction, generation of excessive reactive oxygen species (ROS), and induction of DNA damage (Kochian et al. 2005; Jones et al. 2006; Singh et al. 2017; Jaskowiak et al. 2018; Kocjan et al. 2024). Given that approximately half of the world's potentially arable land is acidic (Zhu and Shen 2023), it is of paramount importance to gain a comprehensive understanding of the tolerance mechanisms to Al toxicity in order to improve crop yields.

Al exclusion from the root apex and internal Al detoxification mechanisms are the two main strategies in plants that underlie resistance to Al toxicity (Kochian et al. 2015). Al exclusion mechanisms include the release of various organic acid anions, phenolic compounds, mucus substances, and phosphate from roots into the rhizosphere (Wang et al. 2020; Li et al. 2023). In particular, organic acid anions such as citrate, malate, and oxalate chelate Al^3+^ ions, leading to the formation of complexes that are not taken up by plant roots (Ur Rahman et al. 2024). It has been demonstrated that the membrane transporter families MATE and ALMT are implicated in the efflux of citrate and malate, respectively (Wang et al. 2023). Internal detoxification of Al is achieved by modification of the cell wall and sequestration of Al within the vacuole (Kocjan et al. 2024). The transport of UDP-glucose into the apoplast has been shown to modify the cell wall structure, thus limiting the Al-binding capacity in the cell wall (Xu et al. 2018a). In addition, the degree of methylation of cell wall pectin is known to have a direct effect on Al resistance (Wei et al. 2021). In the process of vacuolar Al sequestration, Al^3+^ ions first traverse the root plasma membrane via NIP and/or Nramp transporters and enter the cytosol. These ions then form complexes with organic acids or phenolic compounds, which are finally transported into the vacuole by ABC or aquaporin transporters (Kochian et al. 2015; Zhu and Shen 2023).

Omics approaches, such as transcriptomics, proteomics and metabolomics, either individually or in combination, are increasingly being used to understand the complex biological responses of plants to abiotic and biotic stresses (Roychowdhury et al. 2023). These approaches can simultaneously detect changes in gene expression patterns, protein abundance profiles, and metabolite levels between unstressed and stressed plants, thereby allowing the determination of important metabolic pathways and biological processes affected by stress (Gupta et al. 2023). In recent years, extensive omics studies have been conducted to elucidate the mechanisms involved in Al toxicity tolerance in plants (Wang et al. 2014, 2021a; Xu et al. 2018b; Ma and Lin 2019; Pinto et al. 2021; Xie et al. 2022; Ma et al. 2023; Wu et al. 2023). These studies suggest that transporters, the antioxidant defense system, cell wall structure and metabolism, flavonoid biosynthesis, glutathione metabolism, hormone signaling pathways, lipid metabolism, glycolysis/gluconeogenesis, starch and sucrose metabolism, the TCA cycle, and several transcription factors are commonly altered in Al-exposed plant species (Xu et al. 2018b; Ma and Lin 2019; Wang et al. 2021a; Ma et al. 2023; Wu et al. 2023). In addition, the components of galactose metabolism, glycolysis/gluconeogenesis, and starch and sucrose metabolism were found to be more abundant or overexpressed in Al-tolerant maize and rice cultivars than in Al-sensitive ones (Wang et al. 2014; Pinto et al. 2021; Xie et al. 2022).

Wheat is one of the most important cereal crops, with a total global production of 788.95 million metric tons in 2023/2024 (USDA 2024). Due to its high yield potential and nutritional value, wheat provides 17% of the total caloric intake of the human diet (Ashikari and Ma 2015; Biel et al. 2020). Wheat is also a widely used model organism for understanding the mechanisms of tolerance to Al toxicity in plants (Rahman and Upadhyaya 2021). However, only a few studies have employed a well-designed omics approach, particularly proteomics and transcriptomics, to investigate Al-induced changes in wheat (Yang et al. 2018; Luo et al. 2024). Moreover, most of the above-mentioned omics studies on Al stress in other plant species have not used both sensitive and tolerant cultivars, which limits our ability to decipher the key mechanisms of Al toxicity tolerance. Therefore, in this study, we aimed to determine the Al-related metabolic responses in roots of Al-sensitive (Golia-99) and Al-tolerant (Demir-2000) wheat cultivars using nuclear magnetic resonance (NMR) spectroscopy. We hypothesized that (i) there would be inherent differences in the levels of some metabolites affecting tolerance to excess Al between the two cultivars, and (ii) Al toxicity would cause an overrepresentation of common and distinct metabolic pathways in sensitive and tolerant cultivars. To the best of our knowledge, this is the first untargeted comparative metabolomics analysis exploring the intrinsic and induced metabolic signatures underlying Al toxicty tolerance in wheat.

## Materials and methods

### Study species, growth conditions, and root elongation assay

In this study, seeds of two bread wheat cultivars (*Triticum aestivum* L. cv. Demir-2000 and Golia-99) were used. The seeds were obtained from the General Directorate of Agricultural Enterprises of Türkiye. Our preliminary experiments indicated that Golia-99 and Demir-2000 were Al-sensitive and Al-tolerant, respectively, in terms of root elongation. Therefore, we first aimed to verify the Al tolerance capacity of the studied cultivars under hydroponic growth conditions. In this context, wheat seeds were germinated in sterile plastic trays containing autoclaved paper towels moistened with distilled water for 7 days at 20 °C in the dark. The germinated seedlings were grown hydroponically using a modified nutrient solution (pH: 4.2) as described by Samuels et al. (1997). After 3 days, the wheat seedlings were divided into groups of 20, and the initial root length of each seedling was measured. The seedlings were immediately transferred to fresh nutrient solutions containing 0, 10, 30, and 50 µM AlCl_3_.6H_2_O (pH: 4.2) (Samuels et al. 1997; Ryan et al. 2009). The final root length of each seedling was recorded after 24 h of exposure to the Al treatments. Finally, the root elongation of the seedlings was calculated from the data obtained. A total of 3 replicates of 20 roots were measured per treatment group.

### Sample preparation for NMR spectroscopy

The Demir-2000 and Golia-99 wheat seedlings were grown as described above and treated with 0, 10, and 30 µM AlCl_3_.6H_2_O for 24 h (Yang et al. 2018). Subsequently, wheat roots from each treatment and cultivar combination were harvested and stored at −80 °C. Prior to NMR spectroscopy, three independent 0.3-g of root samples per treatment were lyophilized and ground into a powder. 300 mM phosphate buffer (pH: 7.4) was prepared using deuterium oxide (99.9 atom % D) containing 0.5 mM DSS sodium salt (sodium 3-(trimethylsilyl)-1-propanesulfonate). 650 µL of this solution was then added to each root sample. The mixtures were centrifuged at 18000 × g for 20 min at 15 °C after sonication. Finally, the supernatants of the samples (600 µL) were transferred to 5 mm diameter NMR tubes (Çatav et al. 2018).

### NMR spectroscopy, data acquisition, and processing and analysis of spectra

One-dimensional (1D) ^1^H NMR experiments were carried out on a Bruker Avance III 600 MHz NMR spectrometer equipped with a cryogenic triple resonance probe (5 mm) at the National Magnetic Resonance Facility in Madison, USA. The SampleJet sample changer and the NMRbot interface were used for automated data acquisition. The "zgesgp" pulse program, provided by Bruker, which features excitation sculpting for water suppression, was employed for data collection (Fathi et al. 2017). All data were collected at 298 K (25 °C). A total of 32 scans were accumulated, consisting of 64 K time-domain data points and a spectral width of 8417.5 Hz. A relaxation delay of 2 s was applied between transients.

The processor module of the Chenomx NMR Suite (version 8.6, Edmonton, AB, Canada) was used for NMR data processing. A 0.0 ppm singlet peak from a 0.5 mM DSS standard was used for chemical shift referencing and metabolite quantification. The identification of metabolites was achieved through the utilization of the 600 MHz reference library of the Chenomx NMR Suite. The total spectral area for each spectrum was taken as a reference for scaling metabolite concentrations (Elgin et al. 2023).

### Data analysis

One-way ANOVA and Tukey's HSD test were used to compare root elongation data and levels of identified metabolites among treatment groups. Before performing these analyses, each data set was checked by Anderson–Darling and Bartlett tests to ensure that the data met the requirements for the parametric tests. If normality and homogeneity of variance were not achieved, the data were logarithmically or square root transformed. The significance level was set at 0.05 for all tests.

Partial least squares discriminant analysis (PLS-DA), a multivariate dimensionality reduction method, was employed to evaluate metabolomics data obtained from Al-treated and non-Al-treated root samples of Demir-2000 and Golia-99. Prior to analysis, the data were subjected to cube-root transformation and mean centering. The predictive ability of the model (Q^2^) and the goodness of fit (R^2^) were calculated using the cross-validation method. Finally, variable importance in projection (VIP) scores were estimated to determine the contribution of each metabolite to the PLS-DA model (Elgin et al. 2023).

Quantitative pathway analysis was also performed to ascertain which metabolic pathways were overrepresented in Demir-2000 and Golia-99 roots after Al exposure. In this analysis, 10 and 30 μM Al treatments were compared with the respective control group for each cultivar. In the pathway analyses, the same data transformation and scaling procedures were used as in the previous PLS-DA analysis. Relative-betweenness centrality and global test algorithms were applied for topology and pathway enrichment analyses, respectively, and the *Triticum aestivum* (bread wheat) library was selected from the KEGG database. Statistically significant enriched pathways were accepted as those with a false discovery rate (FDR)-adjusted *p*-value of less than 0.05 (Pang et al. 2024).

R (version 4.2.2) and MetaboAnalyst 6.0 software were used to analyze the metabolomics and root growth data.

## Results

### Root elongation

**Fig. 1:**
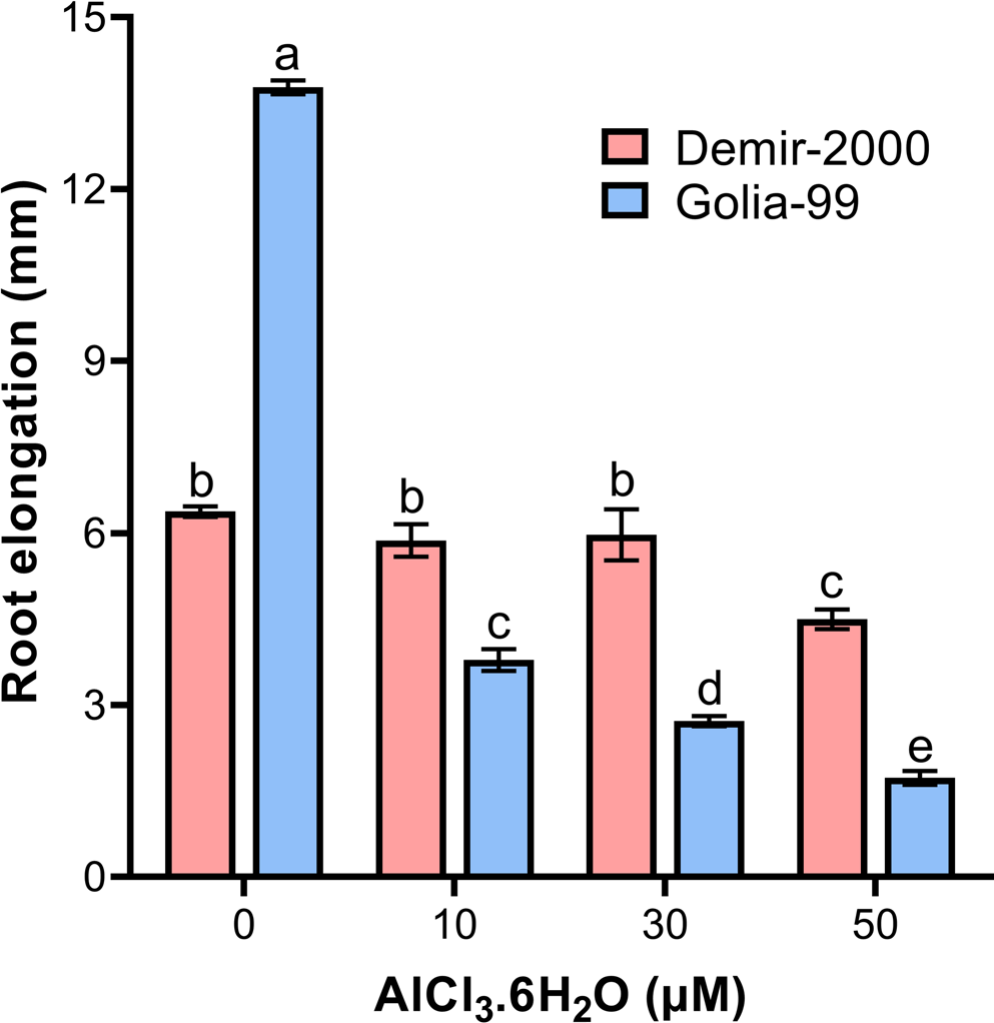
Effects of different concentrations of AlCl_3_.6H_2_O on root elongation (mm) of Al-sensitive (Golia-99) and Al-tolerant (Demir-2000) wheat cultivars. Results are expressed as mean ± SE (n = 3). Different letters on the error bars indicate the significant differences among treatments according to Tukey's HSD test at *p* < 0.05

The effect of different concentrations of AlCl_3_.6H_2_O (hereafter Al) on root elongation of Demir-2000 and Golia-99 for 24 h is shown in Fig. 1. While 50 µM Al significantly reduced the elongation of Demir-2000 roots compared to control conditions, 10 and 30 µM Al treatments had no impact on root growth. On the other hand, all Al treatments resulted in a dramatic decrease in root elongation of Golia-99. For example, the increase in root length of 50 µM Al-treated roots was 12.6% that of non-Al-treated roots (Fig. 1). Overall, the data indicate that Demir-2000 is more tolerant to Al than Golia-99 in terms of root growth.

### Identification of metabolites

**Fig. 2:**
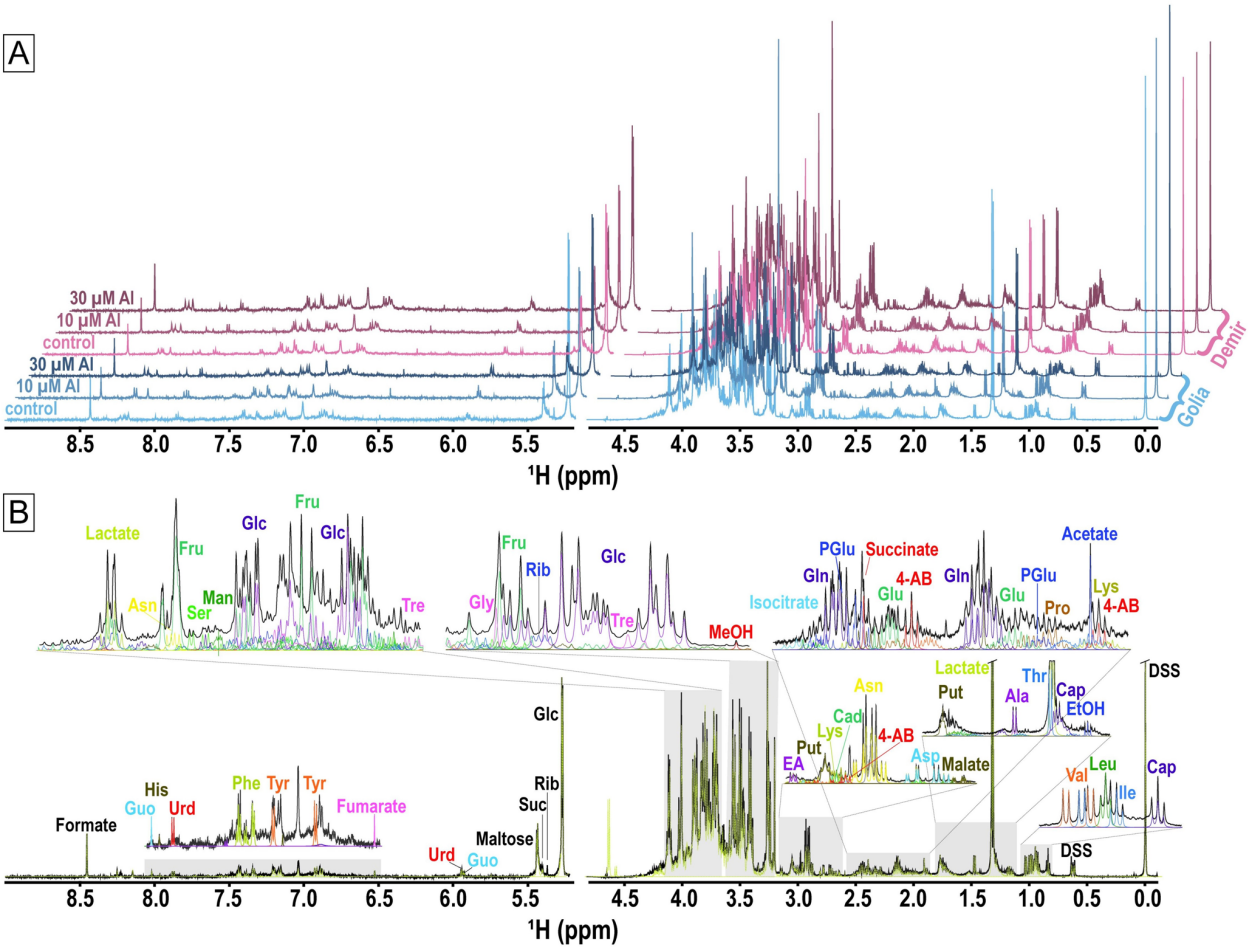
**A** ^1^H NMR spectra of metabolites isolated from wheat cultivars Golia-99 (blue shades) and Demir-2000 (pink shades) treated with 0, 10, and 30 µM Al. The downfield region of the spectrum (5.2 to 9.0 ppm) was amplified 3 times relative to the upfield region (0 to 4.8 ppm) to make the signals more visible. **B** A representative ^1^H NMR spectrum of one of the Golia-99 control replicates. The yellow dotted line shows the sum line for all assigned metabolites. Details of metabolite assignments for the gray-shaded sections of the spectrum are shown in the connected insets. Metabolite names: 4-AB: 4-aminobutyrate, Ala: alanine, Asn: asparagine, Asp: aspartate, Cad: cadaverine, Cap: caprate, EA: ethanolamine, EtOH: ethanol, Fru: fructose, Glc: glucose, Gln: glutamine, Glu: glutamate, Gly: glycine, Guo: guanosine, His: histidine, Ile: isoleucine, Leu: leucine, Lys: lysine, Man: mannose, MeOH: methanol, Phe: phenylalanine, Pro: proline, Put: putrescine, PGlu: pyroglutamate, Rib: ribose, Ser: serine, Suc: sucrose, Thr: threonine, Tre: trehalose, Tyr: tyrosine, Urd: uridine, Val: valine

We identified 54 metabolites from ^1^H NMR spectra of root extracts of Demir-2000 and Golia-99 treated with or without Al using the Chenomx reference library. Figure 2A display the representative NMR spectra of the studied cultivars exposed to 0, 10 and 30 µM Al. Metabolite annotations performed on one of the Golia-99 control spectra are also shown in Fig. 2B. The Metabolomics Workbench database was used to determine the chemical taxonomy of each metabolite (Table S1). The identified metabolites belonged predominantly to the main classes of monosaccharides, fatty acids, amino acids and peptides, and TCA acids.

### Intrinsic differences in metabolite concentrations between cultivars

Our results demonstrated that there was a marked difference between Demir-2000 and Golia-99 roots for the concentration of 15 metabolites under control conditions (Table 1). In particular, the concentrations of several TCA metabolites, such as citrate, isocitrate, and succinate were significantly higher in Demir-2000 than in Golia-99. In addition, 6 of these 15 metabolites were amino acids, and the concentration of 5 of them was higher in Demir-2000 roots (*p* < 0.05). These amino acids included cysteine, glutamate, histidine, lysine, and phenylalanine. Furthermore, the uracil content of Golia-99 roots was about 22% that of Demir-2000 roots. On the other hand, threonine, caprate, fructose, glucose, lactate, and methanol were found in greater concentrations in Golia-99 compared to Demir-2000.

**Table 1:** Effects of 10 and 30 µM AlCl_3_.6H_2_O on metabolite levels in roots of Al-sensitive (Golia-99) and Al-tolerant (Demir-2000) wheat cultivars

Metabolite	Demir-2000			Golia-99		
	0 µM	10 µM	30 µM	0 µM	10 µM	30 µM
4-aminobutyrate	1.00 ± 0.07^b^	1.22 ± 0.03^ab^	1.28 ± 0.06^ab^	1.22 ± 0.06^ab^	1.44 ± 0.08^a^	1.40 ± 0.08^a^
5-aminolevulinate	1.00 ± 0.03^abc^	1.11 ± 0.01^ab^	1.02 ± 0.06^abc^	0.82 ± 0.06^c^	0.88 ± 0.05^bc^	1.18 ± 0.08^a^
Acetate	1.00 ± 0.05^a^	1.28 ± 0.13^a^	0.98 ± 0.04^a^	1.04 ± 0.17^a^	1.12 ± 0.21^a^	0.96 ± 0.04^a^
Alanine	1.00 ± 0.00^a^	0.92 ± 0.05^a^	0.98 ± 0.06^a^	0.97 ± 0.14^a^	1.26 ± 0.21^a^	0.88 ± 0.04^a^
Arginine	1.00 ± 0.06^ab^	0.71 ± 0.06^b^	1.26 ± 0.02^a^	0.77 ± 0.10^b^	0.78 ± 0.09^b^	0.96 ± 0.03^ab^
Asparagine	1.00 ± 0.03^b^	1.20 ± 0.09^ab^	1.53 ± 0.10^a^	1.00 ± 0.15^b^	1.41 ± 0.17^ab^	1.35 ± 0.06^ab^
Aspartate	1.00 ± 0.05^c^	1.65 ± 0.11^a^	1.21 ± 0.03^bc^	0.99 ± 0.12^c^	0.91 ± 0.01^c^	1.38 ± 0.08^ab^
Betaine	1.00 ± 0.02^ab^	1.38 ± 0.08^a^	1.42 ± 0.11^a^	1.00 ± 0.12^ab^	0.86 ± 0.16^b^	0.69 ± 0.05^b^
Cadaverine	1.00 ± 0.18^ab^	0.69 ± 0.05^b^	0.87 ± 0.04^ab^	1.03 ± 0.09^ab^	1.09 ± 0.03^ab^	1.13 ± 0.04^a^
Caprate	1.00 ± 0.03^b^	0.97 ± 0.03^b^	0.98 ± 0.02^b^	2.57 ± 0.24^a^	1.04 ± 0.12^b^	1.40 ± 0.26^b^
Carnitine	1.00 ± 0.11^ab^	1.11 ± 0.20^ab^	1.42 ± 0.11^a^	1.52 ± 0.04^a^	1.10 ± 0.15^ab^	0.76 ± 0.13^b^
Choline	1.00 ± 0.06^a^	1.15 ± 0.03^a^	1.03 ± 0.04^a^	1.16 ± 0.11^a^	1.21 ± 0.16^a^	1.10 ± 0.02^a^
cis-Aconitate	1.00 ± 0.14^a^	0.68 ± 0.04^a^	0.78 ± 0.17^a^	0.70 ± 0.07^a^	0.60 ± 0.10^a^	0.85 ± 0.12^a^
Citrate	1.00 ± 0.01^a^	1.02 ± 0.03^a^	0.93 ± 0.09^a^	0.67 ± 0.01^b^	0.53 ± 0.02^b^	0.59 ± 0.02^b^
Cysteine	1.00 ± 0.11^a^	0.64 ± 0.06^b^	0.71 ± 0.10^ab^	0.51 ± 0.03^b^	0.50 ± 0.00^b^	0.45 ± 0.02^b^
Ethanol	1.00 ± 0.20^a^	0.57 ± 0.02^a^	0.64 ± 0.07^a^	0.62 ± 0.02^a^	0.94 ± 0.19^a^	1.02 ± 0.19^a^
Ethanolamine	1.00 ± 0.05^a^	1.28 ± 0.01^a^	1.17 ± 0.02^a^	1.21 ± 0.16^a^	1.26 ± 0.15^a^	1.21 ± 0.04^a^
Formate	1.00 ± 0.03^b^	1.19 ± 0.04^b^	1.59 ± 0.08^a^	1.33 ± 0.16^ab^	1.22 ± 0.06^ab^	1.30 ± 0.03^ab^
Fructose	1.00 ± 0.02^c^	1.30 ± 0.05^bc^	1.33 ± 0.02^bc^	1.78 ± 0.10^a^	1.42 ± 0.11^ab^	1.36 ± 0.11^bc^
Fumarate	1.00 ± 0.11^a^	0.95 ± 0.03^a^	0.77 ± 0.01^a^	0.81 ± 0.09^a^	0.80 ± 0.07^a^	0.97 ± 0.08^a^
Galactose	1.00 ± 0.03^a^	1.13 ± 0.09^a^	1.10 ± 0.15^a^	0.89 ± 0.02^a^	0.81 ± 0.09^a^	0.91 ± 0.09^a^
Glucarate	1.00 ± 0.04^a^	0.92 ± 0.09^a^	0.80 ± 0.05^a^	0.93 ± 0.03^a^	0.93 ± 0.03^a^	0.88 ± 0.07^a^
Glucose	1.00 ± 0.18^b^	1.45 ± 0.03^ab^	1.36 ± 0.12^ab^	1.63 ± 0.12^a^	1.36 ± 0.05^ab^	1.39 ± 0.12^ab^
Glutamate	1.00 ± 0.01^bc^	1.32 ± 0.11^a^	1.10 ± 0.05^ab^	0.67 ± 0.04^d^	0.79 ± 0.02^ cd^	1.04 ± 0.09^abc^
Glutamine	1.00 ± 0.02^b^	0.99 ± 0.11^b^	1.69 ± 0.09^a^	0.74 ± 0.04^b^	0.74 ± 0.05^b^	0.88 ± 0.08^b^
Glycerol	1.00 ± 0.02^bc^	1.28 ± 0.03^a^	1.26 ± 0.10^ab^	0.87 ± 0.09^ cd^	0.80 ± 0.01^ cd^	0.69 ± 0.03^d^
Glycine	1.00 ± 0.11^a^	1.41 ± 0.24^a^	1.32 ± 0.28^a^	1.67 ± 0.23^a^	0.90 ± 0.11^a^	1.21 ± 0.19^a^
Guanosine	1.00 ± 0.24^ab^	1.21 ± 0.12^a^	0.82 ± 0.11^ab^	0.93 ± 0.16^ab^	0.42 ± 0.05^b^	0.93 ± 0.09^ab^
Histidine	1.00 ± 0.18^a^	0.54 ± 0.01^b^	0.53 ± 0.05^b^	0.33 ± 0.03^b^	0.38 ± 0.02^b^	0.50 ± 0.04^b^
Isocitrate	1.00 ± 0.02^ab^	1.00 ± 0.08^a^	1.07 ± 0.09^a^	0.70 ± 0.03^c^	0.59 ± 0.06^c^	0.73 ± 0.03^bc^
Isoleucine	1.00 ± 0.05^ab^	1.24 ± 0.03^ab^	1.37 ± 0.09^a^	1.12 ± 0.21^ab^	1.01 ± 0.17^ab^	0.68 ± 0.04^b^
Lactate	1.00 ± 0.09^b^	0.67 ± 0.10^b^	0.78 ± 0.10^b^	1.63 ± 0.10^a^	1.13 ± 0.19^ab^	1.03 ± 0.10^b^
Leucine	1.00 ± 0.08^ab^	1.18 ± 0.03^a^	1.19 ± 0.07^a^	0.92 ± 0.19^ab^	0.80 ± 0.10^ab^	0.64 ± 0.02^b^
Lysine	1.00 ± 0.16^a^	0.77 ± 0.05^ab^	0.75 ± 0.05^ab^	0.57 ± 0.04^b^	0.57 ± 0.10^b^	0.56 ± 0.05^b^
Malate	1.00 ± 0.04^c^	1.36 ± 0.09^b^	1.38 ± 0.01^b^	1.10 ± 0.04^bc^	0.89 ± 0.09^c^	1.77 ± 0.11^a^
Maltose	1.00 ± 0.04^ab^	1.18 ± 0.21^ab^	1.47 ± 0.12^a^	0.86 ± 0.08^b^	1.08 ± 0.12^ab^	0.89 ± 0.04^b^
Mannose	1.00 ± 0.05^a^	0.97 ± 0.05^a^	1.12 ± 0.18^a^	0.84 ± 0.08^a^	0.80 ± 0.02^a^	0.77 ± 0.06^a^
Methanol	1.00 ± 0.20^c^	2.46 ± 0.21^a^	1.42 ± 0.15^bc^	1.81 ± 0.08^ab^	1.59 ± 0.08^bc^	1.74 ± 0.10^b^
Methionine	1.00 ± 0.04^ab^	1.41 ± 0.04^a^	1.23 ± 0.07^ab^	0.94 ± 0.11^ab^	0.78 ± 0.15^b^	1.15 ± 0.12^ab^
Phenylalanine	1.00 ± 0.05^a^	0.96 ± 0.05^a^	1.00 ± 0.04^a^	0.69 ± 0.07^b^	0.77 ± 0.07^ab^	0.57 ± 0.02^b^
Proline	1.00 ± 0.00^abc^	0.91 ± 0.07^abc^	1.21 ± 0.08^a^	0.74 ± 0.03^c^	1.11 ± 0.10^ab^	0.88 ± 0.02^bc^
Putrescine	1.00 ± 0.05^b^	1.55 ± 0.03^a^	1.52 ± 0.10^a^	1.11 ± 0.12^b^	1.08 ± 0.10^b^	1.07 ± 0.07^b^
Pyroglutamate	1.00 ± 0.05^b^	1.31 ± 0.02^a^	1.42 ± 0.05^a^	1.00 ± 0.04^b^	0.87 ± 0.02^b^	0.98 ± 0.04^b^
Pyruvate	1.00 ± 0.24^a^	0.82 ± 0.10^a^	0.70 ± 0.11^a^	0.83 ± 0.06^a^	0.91 ± 0.09^a^	0.77 ± 0.06^a^
Ribose	1.00 ± 0.08^a^	1.40 ± 0.02^a^	1.01 ± 0.25^a^	1.38 ± 0.14^a^	1.19 ± 0.02^a^	1.10 ± 0.09^a^
Serine	1.00 ± 0.02^a^	0.90 ± 0.06^a^	1.04 ± 0.03^a^	0.87 ± 0.04^a^	1.03 ± 0.04^a^	0.93 ± 0.11^a^
Succinate	1.00 ± 0.02^a^	0.46 ± 0.03^c^	0.45 ± 0.05^c^	0.63 ± 0.05^bc^	0.80 ± 0.07^ab^	0.73 ± 0.08^b^
Sucrose	1.00 ± 0.17^a^	1.31 ± 0.04^a^	0.90 ± 0.16^a^	0.99 ± 0.11^a^	1.09 ± 0.16^a^	0.90 ± 0.08^a^
Threonine	1.00 ± 0.01^b^	0.88 ± 0.06^b^	1.07 ± 0.03^b^	1.44 ± 0.05^a^	1.15 ± 0.09^ab^	1.03 ± 0.12^b^
Trehalose	1.00 ± 0.22^a^	1.42 ± 0.10^a^	1.07 ± 0.20^a^	1.16 ± 0.03^a^	1.11 ± 0.13^a^	0.99 ± 0.12^a^
Tyrosine	1.00 ± 0.09^abc^	1.10 ± 0.09^ab^	1.22 ± 0.04^a^	0.90 ± 0.06^bc^	0.89 ± 0.07^bc^	0.72 ± 0.01^c^
Uracil	1.00 ± 0.01^a^	0.56 ± 0.03^b^	0.45 ± 0.04^bc^	0.22 ± 0.01^c^	0.63 ± 0.07^b^	0.43 ± 0.09^bc^
Uridine	1.00 ± 0.02^b^	1.75 ± 0.04^a^	1.48 ± 0.09^ab^	1.19 ± 0.11^b^	1.24 ± 0.19^ab^	1.37 ± 0.11^ab^
Valine	1.00 ± 0.09^a^	1.25 ± 0.07^a^	1.35 ± 0.07^a^	1.23 ± 0.24^a^	1.09 ± 0.20^a^	0.85 ± 0.02^a^

Metabolite concentrations in each row are shown relative to the control group of Demir-2000. Values (mean ± SE, n = 3) with different superscripts in the same line are significantly different from each other according to Tukey's HSD test at *p* < 0.05

### Effect of Al treatments on metabolite concentrations

Al treatments caused remarkable changes in the levels of 15 out of 54 metabolites in Demir-2000 roots compared with the control (Table 1). These changes were largely specific to the Al concentration. However, the levels of histidine, malate, putrescine, pyroglutamate, succinate, and uracil were altered in response to both Al concentrations. A total of 11 metabolite levels were increased by one of the Al treatments (Tables 1 and 2). In contrast, the levels of cysteine, histidine, succinate, and uracil showed a decreasing trend in Al-treated Demir-2000 roots. In Golia-99, Al treatments induced a pronounced effect on the levels of 11 metabolites (Table 2). Most of the alterations in metabolite concentrations were associated with 30 µM Al application. After Al exposure, a drastic reduction in the levels of caprate, carnitine and lactate was observed, while uracil levels increased by 186%. Finally, enhanced accumulation of aspartate, glutamate, and malate was detected in both Al-treated wheat cultivars (Table 2).

**Table 2:** Metabolites whose concentrations are significantly (*p* < 0.05) altered by at least one of the Al treatments in roots of Al-sensitive (Golia-99) or Al-tolerant (Demir-2000) wheat cultivars

Metabolite	Main chemical class	Demir-2000	Golia-99
Glycerol	Alcohols and polyols	+	ns
Asparagine	Amino acids and peptides	+	ns
Aspartate	Amino acids and peptides	+	+
Cysteine	Amino acids and peptides	–	ns
Glutamate	Amino acids and peptides	+	+
Glutamine	Amino acids and peptides	+	ns
Histidine	Amino acids and peptides	–	ns
Proline	Amino acids and peptides	ns	+
Pyroglutamate	Amino acids and peptides	+	ns
Threonine	Amino acids and peptides	ns	–
Formate	Carboxylic acids	+	ns
Carnitine	Carnitines	ns	–
5-aminolevulinate	Fatty acids	ns	+
Caprate	Fatty acids	ns	–
Putrescine	Fatty amines	+	ns
Fructose	Monosaccharides	ns	–
Methanol	Primary alcohols	+	ns
Uracil	Pyrimidines	–	+
Uridine	Pyrimidines	+	ns
Lactate	Short-chain acids	ns	–
Malate	TCA acids	+	+
Succinate	TCA acids	–	ns

" + " and "**–**" indicate increases and decreases, respectively, in metabolite concentrations after Al treatment. "ns" denotes an insignificant change in the concentration of a metabolite upon exposure to Al

**Fig. 3:**
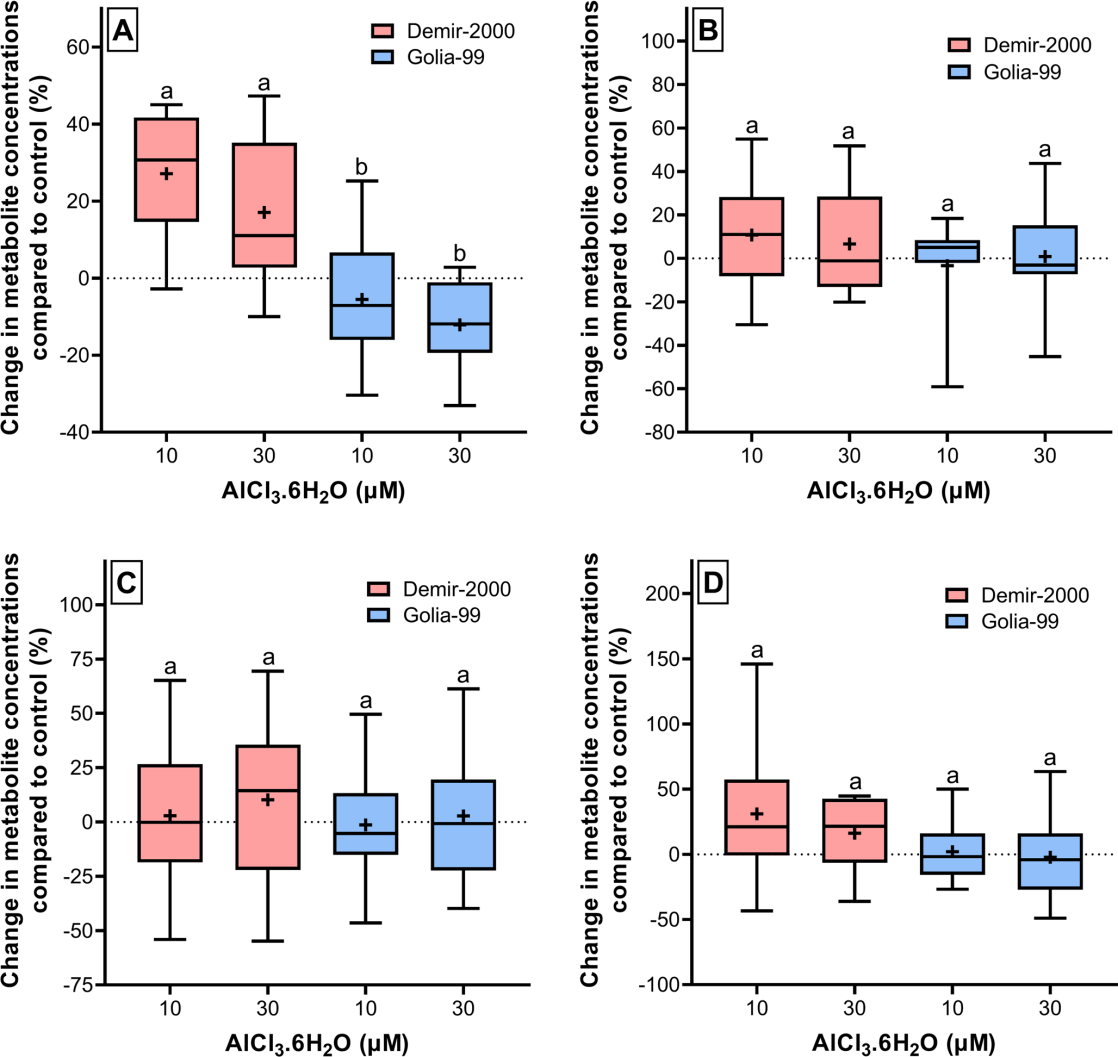
Changes in the concentrations of metabolites associated with **A** carbohydrates, **B** fatty acyls, **C** organic acids, and **D** organic oxygen and nitrogen compounds in Al-treated wheat roots compared to control conditions. The " + " and "straight line" in the boxes represent the mean and median values, respectively. Different letters on the upper whiskers indicate the significant differences among treatments according to Tukey's HSD test at *p* < 0.05

In this study, we also evaluated the effect of Al treatments on chemical super classes, such as carbohydrates, fatty acyls, and organic acids, into which most of the metabolites are classified (Table S1, Fig. 3). The levels of metabolites classified as carbohydrates in Demir-2000 roots increased by an average of 27.2% and 17.1% after 10 and 30 µM Al treatments, respectively. Conversely, the levels of these metabolites decreased by an average of 5.5% and 12.1%, respectively, in Golia-99 roots after the same treatments (Fig. 3A). One-way ANOVA also showed a significant difference in the change of carbohydrate levels between Demir-2000 and Golia-99 upon Al treatments (*p* < 0.05). The concentrations of metabolites classified as fatty acyls in Al-treated Demir-2000 and Golia-99 differed from control conditions by a range of − 3.4% to 10.8% with no notable difference between cultivars (Fig. 3B). A very similar trend was observed for metabolites belonging to the group of organic acids (Fig. 3C). Lastly, 10 and 30 µM Al increased the levels of metabolites associated with organic oxygen and nitrogen compounds by an average of 31.0% and 16.2%, respectively, in Demir-2000. However, no significant difference was seen between the two cultivars exposed to Al in terms of changes in metabolite levels (Fig. 3D).

### PLS-DA analysis

**Fig. 4:**
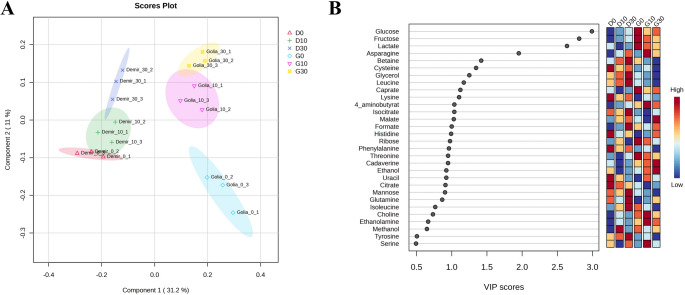
**A** PLS-DA scores plot of metabolomics data from Golia-99 (Al-sensitive) and Demir-2000 (Al-tolerant) wheat roots treated with 0, 10, and 30 µM AlCl_3_·6H_2_O. **B** VIP scores plot resulting from the model. The heat map on the right side of the plot shows the relative concentrations of metabolites in the roots of wheat cultivars exposed to Al treatments. Metabolites with a VIP score greater than 1.0 are considered important for group differentiation. (D0: Demir-2000 - 0 µM Al, D10: Demir-2000 - 10 µM Al, D30: Demir-2000 - 30 µM Al, G0: Golia-99 - 0 µM Al, G10: Golia-99 - 10 µM Al, G30: Golia-99 - 30 µM Al)

The scores plot and VIP scores obtained from the PLS-DA are shown in Fig. 4. The predictive ability of the model (Q^2^ value) and the goodness of fit (R^2^ value) are calculated as 0.93 and 0.98, respectively. The results revealed that the metabolic profiles of Demir-2000 and Golia-99 were distinctly different under control conditions (Fig. 4A). In Demir-2000, a clear separation was observed between the control and 30 µM Al treatment groups, while the 10 µM Al treatment group overlapped with both. In Golia-99, both Al treatments grouped apart from the control, indicating a metabolic differentiation between Al-treated and untreated wheat roots. In addition, variable importance in the projection (VIP) scores were calculated to evaluate the contribution of each variable (metabolite) in the projection of the PLS-DA model (Fig. 4B). Glucose, fructose, lactate, asparagine, betaine, and cysteine were the major metabolites with the greatest influence on the model.

### Pathway analysis

**Fig. 5:**
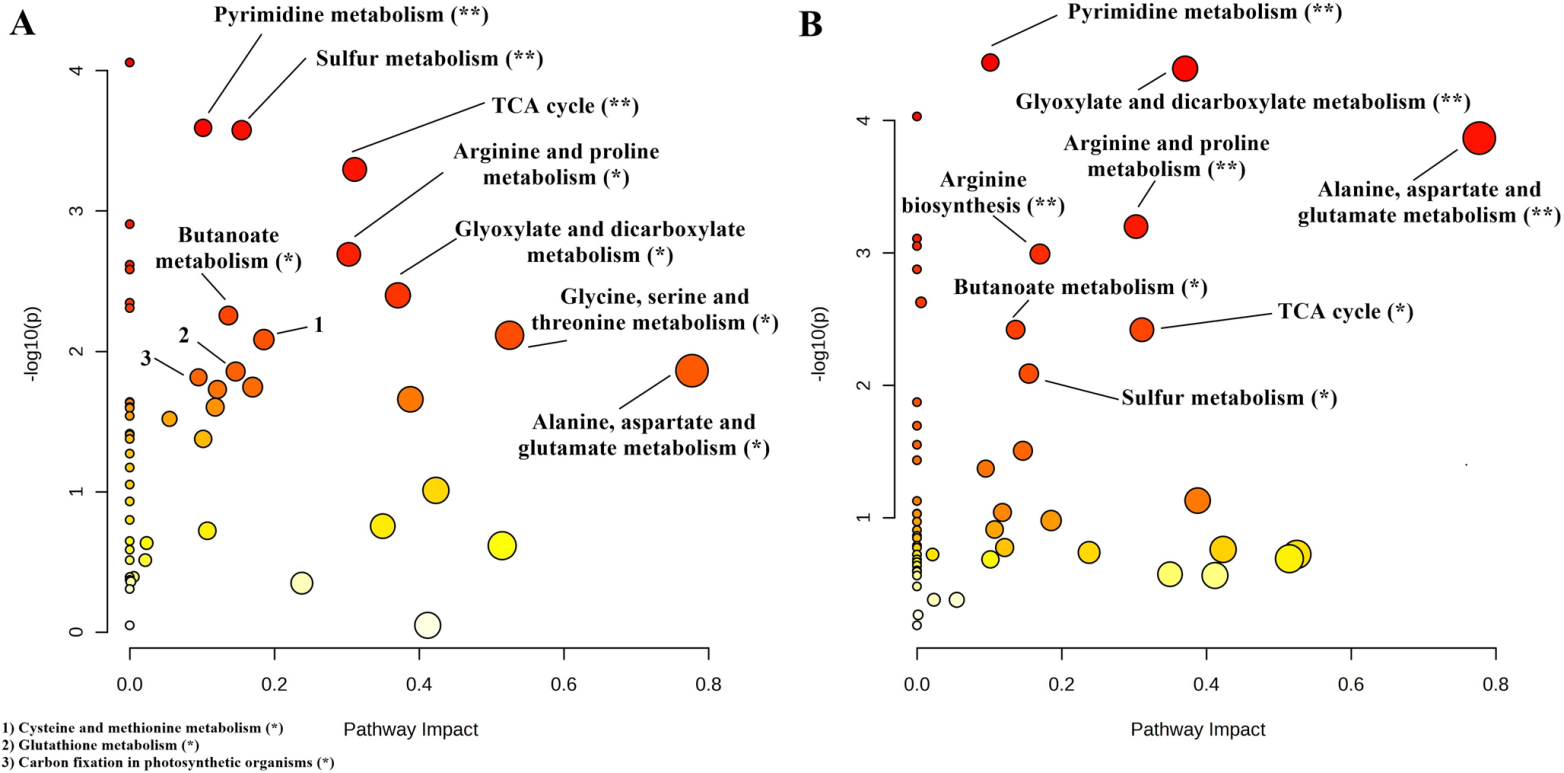
Effects of **A** 10 and **B** 30 µM AlCl_3_.6H_2_O on metabolic pathways in Al-tolerant Demir-2000 wheat roots. The "significance level" and "pathway impact" of the analysis are represented by circles. While the color direction from yellow to orange to red indicates the higher −log10(p), the size of the circles implies the impact of the pathway. (ns: not significant; *p < 0.05; ∗ *p* < 0.01)

**Fig. 6:**
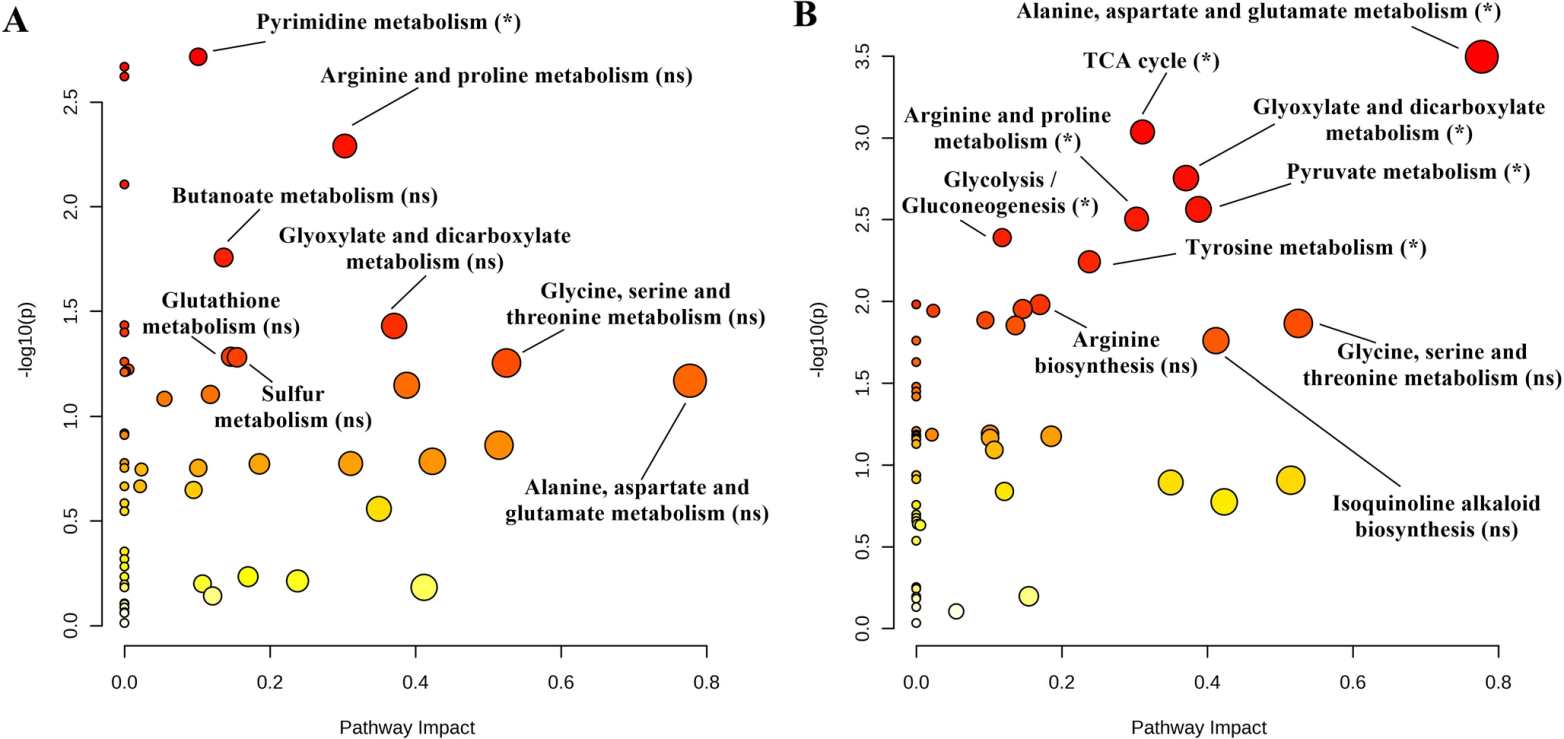
Effects of **A** 10 and **B** 30 µM AlCl_3_.6H_2_O on metabolic pathways in Al-sensitive Golia-99 wheat roots. The "significance level" and "pathway impact" of the analysis are represented by circles. While the color direction from yellow to orange to red indicates the higher −log10(p), the size of the circles implies the impact of the pathway. (ns: not significant; *p < 0.05; ∗*p* < 0.01)

**Fig. 7:**
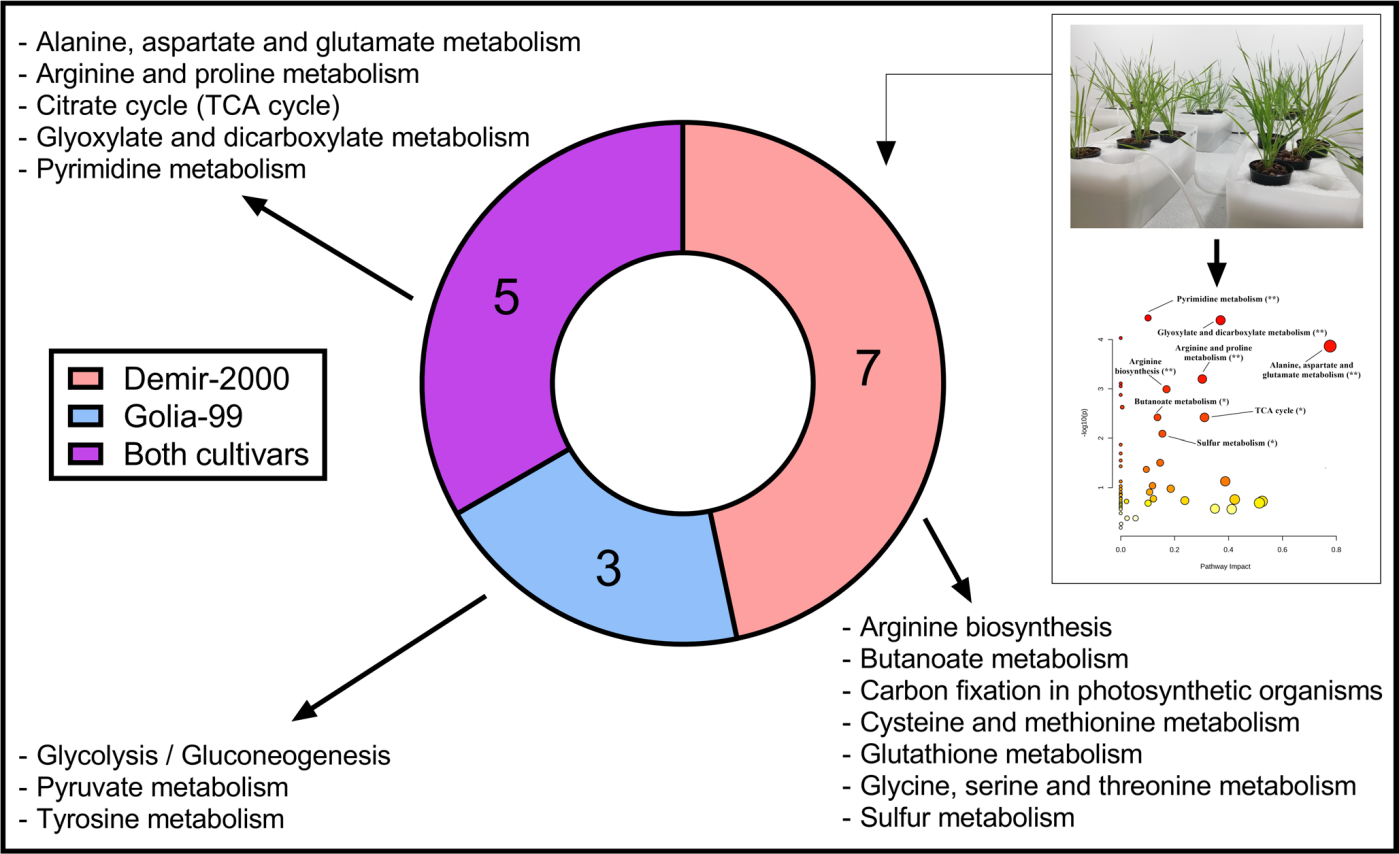
Schematic representation of the metabolic pathways affected by at least one of the Al treatments in roots of Al-sensitive (Golia-99) and Al-tolerant (Demir-2000) wheat cultivars. The upper right part of the figure is the graphical summary of the quantitative pathway analysis in Al-treated wheat seedlings

The quantitative pathway analysis of Demir-2000 and Golia-99 roots subjected to 10 and 30 µM AlCl_3_.6H_2_O is presented in Figs. 5, 6 and 7 and Tables S2-S5. Arginine and proline, pyrimidine, sulfur, butanoate, alanine, aspartate and glutamate, glyoxylate and dicarboxylate metabolisms, and TCA cycle were affected by both Al treatments in Demir-2000 roots (Fig. 5A and B). On the other hand, carbon fixation in photosynthetic organisms, arginine biosynthesis, as well as glycine, serine and threonine, cysteine and methionine, and glutathione metabolisms were altered depending on Al concentration. In Golia-99, 10 µM Al influenced only pyrimidine metabolism, whereas seven metabolic pathways, including pyruvate metabolism, glycolysis/gluconeogenesis, and tyrosine metabolism responded to 30 µM Al (Fig. 6A and B). Finally, alanine, aspartate and glutamate, arginine and proline, glyoxylate and dicarboxylate, pyrimidine metabolisms, and the TCA cycle were found to be affected by at least one Al treatment in both cultivars (Fig. 7).

## Discussion

Aluminum (Al) toxicity is a major constraint on crop growth and yield in acidic soils (Munyaneza et al. 2024). Al stress has been shown to affect many physiological processes in plants, including root elongation, cell wall polysaccharide composition, and oxidative status (Wang et al. 2021b; Yu et al. 2023). On the other hand, there is a considerable variation in tolerance to Al toxicity between and within species (Kocjan et al. 2024). It has been documented that the ability of plant species for organic acid and phenolic compound-mediated exclusion and/or vacuolar sequestration of Al is a crucial mechanism underlying the observed variability in Al tolerance (Kochian et al. 2015). Changes in cell wall metabolism and antioxidant defense responses also provide another explanation for the intraspecific variation in tolerance to Al (Xu et al. 2022b). However, the similarities and differences in the metabolic responses of cultivars in terms of Al sensitivity remain poorly understood, although some insights have been provided by Xie et al. (2022) and Wang et al. (2023). To address this knowledge gap, here we determined the root metabolic profiles of Al-sensitive (Golia-99) and Al-tolerant (Demir-2000) wheat cultivars using NMR spectroscopy. The current results reveal that Al toxicity affects five metabolic pathways in both Golia-99 and Demir-2000 roots. These include alanine, aspartate and glutamate metabolism, pyrimidine metabolism, glyoxylate and dicarboxylate metabolism, the TCA cycle, and arginine and proline metabolism. Moreover, Al stress resulted in a significant alteration in several pathways in a cultivar-specific manner, indicating that particular metabolic modifications may be involved in the regulation of Al tolerance. Lastly, two cultivars showed differentiation with respect to the intrinsic concentrations of 15 metabolites.

Differences in constitutive levels of chemical compounds between cultivars and closely related species have been suggested to play a role in intra- and interspecies variability in tolerance to abiotic stressors, including Al toxicity (Chen et al. 2020; Cárcamo-Fincheira et al. 2021). For example, the root ascorbate content of Al-resistant blueberry cultivars (Cargo and Camellia) was found to be 2–4 times higher than that of the Al-sensitive blueberry cultivar Star under normal growth conditions (Cárcamo-Fincheira et al. 2021). In addition, Chen et al. (2020) observed that the activities of enzymes associated with polyphenol biosynthesis in lettuce were inherently lower in the Al-sensitive genotype compared to the Al-tolerant one. In this study, the levels of amino acids such as cysteine, glutamate, histidine, lysine, and phenylalanine were 45–203% greater in Demir-2000 roots than in Golia-99 roots under control conditions. These amino acids are known to be essential for many metabolic processes and stress tolerance in plants (Trovato et al. 2021). For instance, phenylalanine is a precursor of phenylpropanoids, like phenolic acids, flavonoids, anthocyanins, condensed tannins, and lignins (Kumari et al. 2023). It has been shown that phenylpropanoid metabolism and phenolic biosynthesis are up-regulated in Al-exposed plants (Ma and Lin 2019; Chen et al. 2020; Ling et al. 2023; Ma et al. 2023). Based on these findings, it can be postulated that genotypes or species with high phenylalanine levels may exhibit greater tolerance to Al toxicity than those with low phenylalanine levels. Furthermore, glutamate and cysteine are key components in the biosynthesis of glutathione (Hasanuzzaman et al. 2017), which is thought to play a pivotal role in protecting plants from oxidative damage induced by Al toxicity (Silva 2012; Xie et al. 2022). Previous omics studies have also demonstrated that excess Al can modify glutathione metabolism in wheat, *Citrus sinensis*, and *Lemna minor* (Yang et al. 2018; Su et al. 2019; Wu et al. 2022). Consistent with the aforementioned findings, the present results indicate that Al toxicity affects the glutathione metabolism in Al-tolerant Demir-2000 roots. Taken together, naturally higher levels of glutamate and cysteine may confer Al tolerance via the induction of glutathione metabolism.

Tricarboxylic acid (TCA) cycle metabolites (e.g., citrate and malate), which belong to the superclass of organic acids, play a central role not only in cellular energy production but also in plant resistance to Al toxicity (Zhang et al. 2023). These organic acid anions are involved in both Al exclusion and internal tolerance/detoxification processes by chelating Al^3+^ ions (Kochian et al. 2015; Ur Rahman et al. 2024). In wheat, Al resistance has been shown to be highly correlated with root malate efflux (Kopittke et al. 2017). Root citrate efflux is also important for Al resistance in some wheat cultivars, such as Carazinho and Toropi (Ryan et al. 2009). In this work, we determined the concentrations of several TCA metabolites in roots of Al-sensitive and -tolerant wheat cultivars under control and Al stress conditions. Our results reveal that the levels of citrate, isocitrate, and succinate in Demir-2000 roots are intrinsically higher (43–59%) than those in Golia-99 roots. This difference in TCA metabolite levels may be a contributing factor to the superior Al tolerance of Demir-2000 compared to Golia-99. In addition, in both cultivars, at least one Al treatment caused a significant increase in root malate content and exerted a pronounced effect on the TCA cycle. Overall, the current results align with those of previous research demonstrating that excess Al can increase root malate and/or citrate levels (Ikka et al. 2013; Donnelly et al. 2024) and alter the expression of genes and proteins associated with the TCA cycle in various plants (Liu et al. 2017; Su et al. 2019; Wu et al. 2023).

**Fig. 8:**
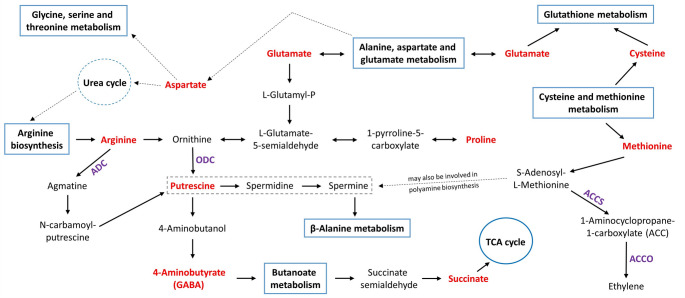
A simplified overview of arginine and proline metabolism and its interacting pathways. The blue rectangles and circles indicate the metabolic pathways, while the purple font represents the enzymes. The metabolites shown in red were identified in this study. The scheme was generated according to the KEGG module database, Majumdar et al. (2016), and Xu et al. (2022a). *ADC* Arginine decarboxylase; *ODC* Ornithine decarboxylase; *ACCS* ACC synthase; *ACCO* ACC oxidase

Polyamines, such as putrescine, spermidine, and spermine, are small polycationic molecules involved in the regulation of embryogenesis, seedling establishment, reproductive development, senescence, and abiotic stress tolerance in plants (Blázquez 2024). It has been shown that exogenous application of these polyamines can improve root growth in several plants under Al toxicity (Chen et al. 2008; Wang et al. 2013; Jiang et al. 2021). In addition, endogenous levels of polyamines, especially putrescine, have been observed to be increased by Al stress (Yu et al. 2015; Nahar et al. 2017). The amelioration of Al toxicity by polyamines is attributed to changes in the processes of Al accumulation, oxidative and antioxidative status, ethylene metabolism, and cell wall structure and function (Yu et al. 2016, 2018; Zhu et al. 2019; Ghosh et al. 2024). For example, Zhu et al. (2019) found that putrescine reduced Al accumulation in the cell wall of Al-treated rice seedlings by decreasing pectin and hemicellulose levels as well as pectin methylesterase activity. Moreover, putrescine was suggested to reduce ROS production by inhibiting Al-induced cell wall-bound polyamine oxidase and plasma membrane NADPH activities (Yu et al. 2018). Finally, putrescine has been reported to decrease elevated ethylene levels caused by Al toxicity via the suppression of ethylene biosynthesis-related genes or enzymes (e.g., ACC synthase and ACC oxidase) (Yu et al. 2016; Zhu et al. 2019). In this work, both Al treatments resulted in a significant increase (52% to 55%) in the putrescine level in Demir-2000 roots. However, no such increase was observed in Al-sensitive Golia-99 roots. Furthermore, our results showed that arginine and proline metabolism, arginine biosynthesis, and alanine, aspartate and glutamate metabolism were highly overrepresented in Demir-2000 after Al exposure. These metabolic pathways are known to be engaged in the biosynthesis of various amino acids and polyamines, including proline and putrescine (Fig. 8). In particular, arginine and ornithine decarboxylases are linked to arginine and proline metabolism and are essential for the biosynthesis of putrescine (Majumdar et al. 2016). The activities of these two enzymes have also been demonstrated to be increased in bean, rice, and wheat seedlings subjected to Al toxicity (Wang et al. 2013; Yu et al. 2015; Jiang et al. 2021). It is noteworthy that despite the absence of alterations in putrescine levels and arginine biosynthesis in Golia-99 roots following Al treatments, proline levels in this cultivar were increased by Al toxicity. It can thus be proposed that a switch in the biosynthesis of proline and putrescine, which is regulated by the arginine and proline metabolism, may affect the ability of a species or cultivar to tolerate Al. Taken as a whole, arginine and proline metabolism appears to be a major target of Al toxicity in wheat, and elevated levels of putrescine provide protection against the deleterious effects of excess Al.

Soluble sugars, like trehalose, glucose, and sucrose, are important signaling molecules implicated in the modulation of many metabolic, structural and developmental processes as well as abiotic stress tolerance in plants (Varshney et al. 2023). Depending on the molecule, soluble sugars can improve abiotic stress tolerance through a variety of mechanisms, including membrane stabilization, protein structure protection, ROS scavenging, osmotic potential adjustment, and maintenance of cellular energy status (Keunen et al. 2013; Saddhe et al. 2021). Soluble sugar concentrations in plant tissues have been found to increase or decrease in response to excess Al (Zhu et al. 2022; Zhou et al. 2024). In addition, several studies have documented that Al toxicity alters galactose metabolism and starch and sucrose metabolism in rice and *Citrus sinensis* (Wu et al. 2022; Xie et al. 2022; Wang et al. 2023). Some proteomics investigations have also shown a remarkable increase in sucrose synthase activity in Al-treated rice and maize plants, especially in those of Al-tolerant cultivars (Wang et al. 2014; Pinto et al. 2021). The present research did not provide evidence that Al treatments have a direct effect on the above-mentioned sugar-related pathways. Furthermore, there were almost no significant fluctuations in the concentrations of individual monosaccharides or disaccharides in both cultivars after Al exposure. Nevertheless, a discernible pattern emerged when all sugars identified through NMR spectroscopy were classified together (Fig. 3A). In this context, Al treatments led to an increase in the levels of these sugars in Demir-2000 roots with an average of 17.1–27.2%. In contrast, the levels of soluble sugars in Golia-99 roots were reduced by an average of 5.5–12.1% under Al treatments. These findings, combined with those from previous studies, suggest that changes in carbohydrate-related pathways and soluble sugar concentrations may potentially influence the ability of plants to tolerate Al toxicity. It is also worth mentioning that the glucose, fructose, and total soluble sugar contents of non-Al-treated Golia-99 roots were 63%, 78%, and 19% higher, respectively, than those of non-Al-treated Demir-2000 roots (Table 1, Fig. 4B). Given that high sugar status in plants is often associated with accelerated growth (Rosa et al. 2009), it can be speculated that the enhanced root growth observed in Golia-99 relative to Demir-2000 under control conditions may be due to the different sugar levels possessed by the two cultivars (Fig. 1).

The accumulation of amino acids and their derivatives in plant tissues in response to abiotic stress conditions has been widely reported in the literature (Ali et al. 2019; Trovato et al. 2021; and references therein). In this study, we found that Al toxicity elevated the concentrations of aspartate and glutamate in both cultivars. However, the levels of asparagine, glutamine, and pyroglutamate were increased exclusively in Demir-2000 roots. These amino acids play a crucial role in improving plant stress tolerance through a range of mechanisms. For instance, some abiotic stresses, including excess Al, have been shown to enhance protein degradation, which in turn leads to ammonium accumulation and its subsequent toxicity. In this regard, the amides asparagine and glutamine are proposed to counteract ammonium toxicity and facilitate the reassimilation of released nitrogen (Herrera-Rodríguez et al. 2007; Navascués et al. 2012; Miranda et al. 2016). In addition, pyroglutamate is known to be function as a glutamate reservoir and to be related to glutathione metabolism and protein turnover (Barding et al. 2013; Zhao et al. 2018). Pyroglutamate has also been shown to be involved in 5-aminolevulinic acid-mediated salt stress alleviation in creeping bentgrass (Yang et al. 2014). Moreover, a decline in pyroglutamate levels is considered as an indicator of increased oxidative stress in plants (Yu et al. 2024). Collectively, our results suggest that the accumulation of asparagine, glutamine, and pyroglutamate amino acids may enhance the tolerance of wheat plants to Al toxicity.

**Fig. 9:**
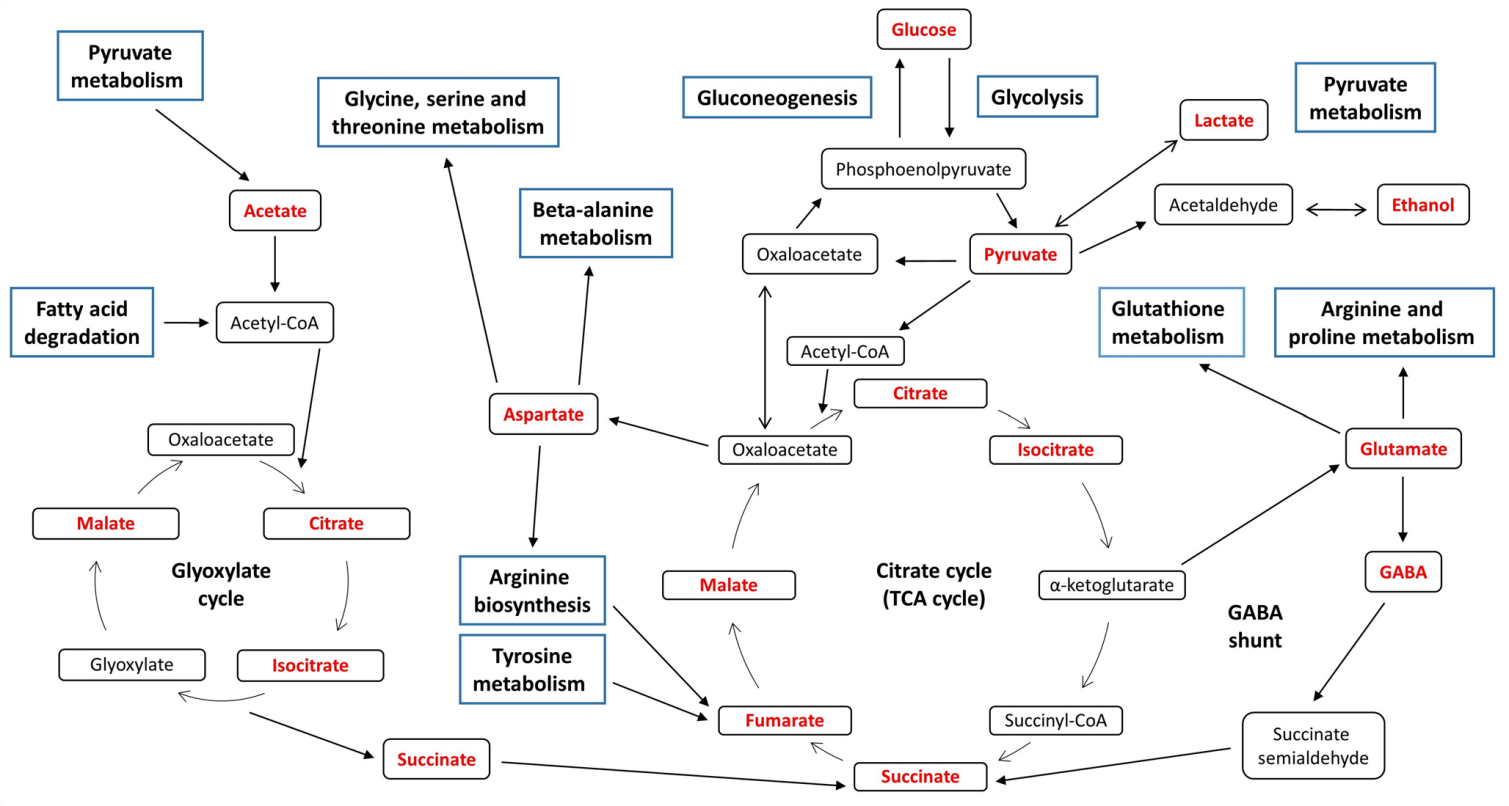
A simplified overview of the glyoxylate cycle, glycolysis/gluconeogenesis, pyruvate metabolism, and the TCA cycle and their interacting pathways. The blue rectangles indicate the metabolic pathways. The metabolites shown in red were identified in this study. The scheme was generated according to the KEGG module database and Nakatsukasa et al. (2015)

Tyrosine metabolism is implicated in the synthesis of molecules, such as ubiquinone, plastoquinone, and tocopherols, which are associated with the respiratory chain, electron transport through the thylakoid membrane, and antioxidant defense, respectively (Xu et al. 2020). Tyrosine is also a precursor of various secondary metabolites (e.g., benzylisoquinoline alkaloids, betalains, and p-coumaric acid), and its degradation product, fumarate, is incorporated into the TCA cycle (Schenck and Maeda 2018). In this work, Al toxicity caused an overrepresentation of tyrosine metabolism only in Golia-99 roots. Two recent studies also demonstrated that Al treatments enriched tyrosine metabolism in both highly Al-sensitive and -resistant olive germplasm, as well as in an Al-sensitive hydrangea cultivar (Chen et al. 2022; Niu et al. 2022). Thus, tyrosine metabolism seems to be an important pathway influencing the tolerance capacity to Al stress in several plant species. Furthermore, we found that glyoxylate and dicarboxylate metabolism and the TCA cycle were overrepresented in both cultivars, whereas glycolysis/gluconeogenesis and pyruvate metabolism were only overrepresented in the roots of the Golia-99 cultivar. These four interrelated pathways provide essential support for a multitude of metabolic processes (Fig. 9), including maintenance of energy homeostasis, carbohydrate metabolism, amino acid biosynthesis, and fatty acid degradation (Nakatsukasa et al. 2015; Walker et al. 2021). The results as a whole indicate that Al toxicity has a profound effect on most of the primary pathways related to central carbohydrate and energy metabolism in Golia-99 roots. Finally, quantitative pathway analysis revealed that glycine, serine and threonine metabolism, sulfur metabolism, and cysteine and methionine metabolism were only overrepresented in Al-treated Demir-2000 roots. These interconnected pathways are responsible for the biosynthesis of sulfur-containing compounds, like hydrogen sulfide, glutathione, S-adenosyl-L-methionine, and phytochelatins, which play a critical role in plant defense against abiotic stresses (Capaldi et al. 2015; Rosa-Téllez et al. 2023).

## Conclusion

Our study showed that there were significant differences in the levels of many metabolites between Golia-99 and Demir-2000 roots under control and Al stress conditions. In particular, the concentrations of metabolites belonging to the amino acid and TCA groups, such as glutamate, cysteine, phenylalanine, citrate, isocitrate, and succinate were naturally higher in Demir-2000 than in Golia-99. In addition, the accumulation of putrescine, soluble sugars, and several amino acids (e.g., asparagine, glutamine, and pyroglutamate) was observed in Demir-2000 roots in response to Al toxicity. Moreover, specific patterns of representation in amino acid, carbohydrate, and energy-related pathways induced by Al appear to be of great importance in explaining the difference in Al tolerance between Golia-99 and Demir-2000. In light of our findings, the following strategies may be employed to improve Al tolerance in wheat through breeding and genetic manipulation: (i) the over-expression of regulatory genes associated with pathways that are overrepresented in Al-tolerant cultivars (e.g., the TCA cycle and glutathione metabolism), (ii) increasing the biosynthesis or limiting the catabolism of molecules involved in Al tolerance, such as putrescine and glutamine, and (iii) determining potential biomarkers for Al stress (e.g. aspartate and glutamate). Further research on Al-induced metabolic changes in these wheat cultivars during subsequent developmental stages is necessary in order to draw significant conclusions for practical applications.

## Supplementary Information

Below is the link to the electronic supplementary material.

Supplementary file1 (DOCX 1949 kb)

## Data Availability

Data will be made available on request.
