# Supplementary material for: Intrinsic and induced metabolic signatures underpin aluminum tolerance in bread wheat: a comparative metabolomics approach
Source: Physiol Mol Biol Plants. 2025 Jul 22;31(6):1011–26. doi: 10.1007/s12298-025-01622-1 (PMC12314282; doi:10.1007/s12298-025-01622-1)

**Tables**

**Table S1.** Chemical taxonomy of metabolites identified from NMR spectra of Al-sensitive Golia-99 and Al-tolerant Demir-2000 roots.

| **Metabolite** | **Super class** | **Main class** | **Sub class** |
| --- | --- | --- | --- |
| Maltose | Carbohydrates | Disaccharides | Disaccharides |
| Sucrose | Carbohydrates | Disaccharides | Disaccharides |
| Trehalose | Carbohydrates | Disaccharides | Disaccharides |
| Fructose | Carbohydrates | Monosaccharides | Hexoses |
| Galactose | Carbohydrates | Monosaccharides | Hexoses |
| Glucose | Carbohydrates | Monosaccharides | Hexoses |
| Mannose | Carbohydrates | Monosaccharides | Hexoses |
| Ribose | Carbohydrates | Monosaccharides | Pentoses |
| 4-aminobutyrate | Fatty Acyls | Fatty acids | Amino FA |
| 5-aminolevulinate | Fatty Acyls | Fatty acids | Amino FA |
| Glucarate | Fatty Acyls | Fatty acids | Dicarboxylic acids |
| Acetate | Fatty Acyls | Fatty acids | Saturated FA |
| Caprate | Fatty Acyls | Fatty acids | Saturated FA |
| Cadaverine | Fatty Acyls | Fatty amines | Monoalkylamines |
| Putrescine | Fatty Acyls | Fatty amines | Monoalkylamines |
| Guanosine | Nucleic acids | Purines | Purine ribonucleosides |
| Uracil | Nucleic acids | Pyrimidines | Other pyrimidines |
| Uridine | Nucleic acids | Pyrimidines | Pyrimidine ribonucleosides |
| Alanine | Organic acids | Amino acids and peptides | Amino acids |
| Arginine | Organic acids | Amino acids and peptides | Amino acids |
| Asparagine | Organic acids | Amino acids and peptides | Amino acids |
| Aspartate | Organic acids | Amino acids and peptides | Amino acids |
| Betaine | Organic acids | Amino acids and peptides | Amino acids |
| Cysteine | Organic acids | Amino acids and peptides | Amino acids |
| Glutamate | Organic acids | Amino acids and peptides | Amino acids |
| Glutamine | Organic acids | Amino acids and peptides | Amino acids |
| Glycine | Organic acids | Amino acids and peptides | Amino acids |
| Histidine | Organic acids | Amino acids and peptides | Amino acids |
| Isoleucine | Organic acids | Amino acids and peptides | Amino acids |
| Leucine | Organic acids | Amino acids and peptides | Amino acids |
| Lysine | Organic acids | Amino acids and peptides | Amino acids |
| Methionine | Organic acids | Amino acids and peptides | Amino acids |
| Phenylalanine | Organic acids | Amino acids and peptides | Amino acids |
| Proline | Organic acids | Amino acids and peptides | Amino acids |
| Pyroglutamate | Organic acids | Amino acids and peptides | Amino acids |
| Serine | Organic acids | Amino acids and peptides | Amino acids |
| Threonine | Organic acids | Amino acids and peptides | Amino acids |
| Tyrosine | Organic acids | Amino acids and peptides | Amino acids |
| Valine | Organic acids | Amino acids and peptides | Amino acids |
| Formate | Organic acids | Carboxylic acids | Carboxylic acids |
| Lactate | Organic acids | Short-chain acids | Short-chain acids |
| Pyruvate | Organic acids | Short-chain acids | Short-chain acids |
| cis-Aconitate | Organic acids | TCA acids | TCA acids |
| Citrate | Organic acids | TCA acids | TCA acids |
| Fumarate | Organic acids | TCA acids | TCA acids |
| Isocitrate | Organic acids | TCA acids | TCA acids |
| Malate | Organic acids | TCA acids | TCA acids |
| Succinate | Organic acids | TCA acids | TCA acids |
| Ethanolamine | Organic nitrogen compounds | Amines | 1,2-aminoalcohols |
| Carnitine | Organic nitrogen compounds | Carnitines | Carnitines |
| Choline | Organic nitrogen compounds | Cholines | Cholines |
| Glycerol | Organic oxygen compounds | Alcohols and polyols | 1,2-diols |
| Ethanol | Organic oxygen compounds | Primary alcohols | Primary alcohols |
| Methanol | Organic oxygen compounds | Primary alcohols | Primary alcohols |

**Table S2.** The results of quantitative pathway analysis for Al-tolerant Demir-2000 roots treated with 10 µM AlCl_3_.6H_2_O. FDR indicates that *p*-values were adjusted using the false discovery rate. The impact of pathways was estimated by topology analysis.


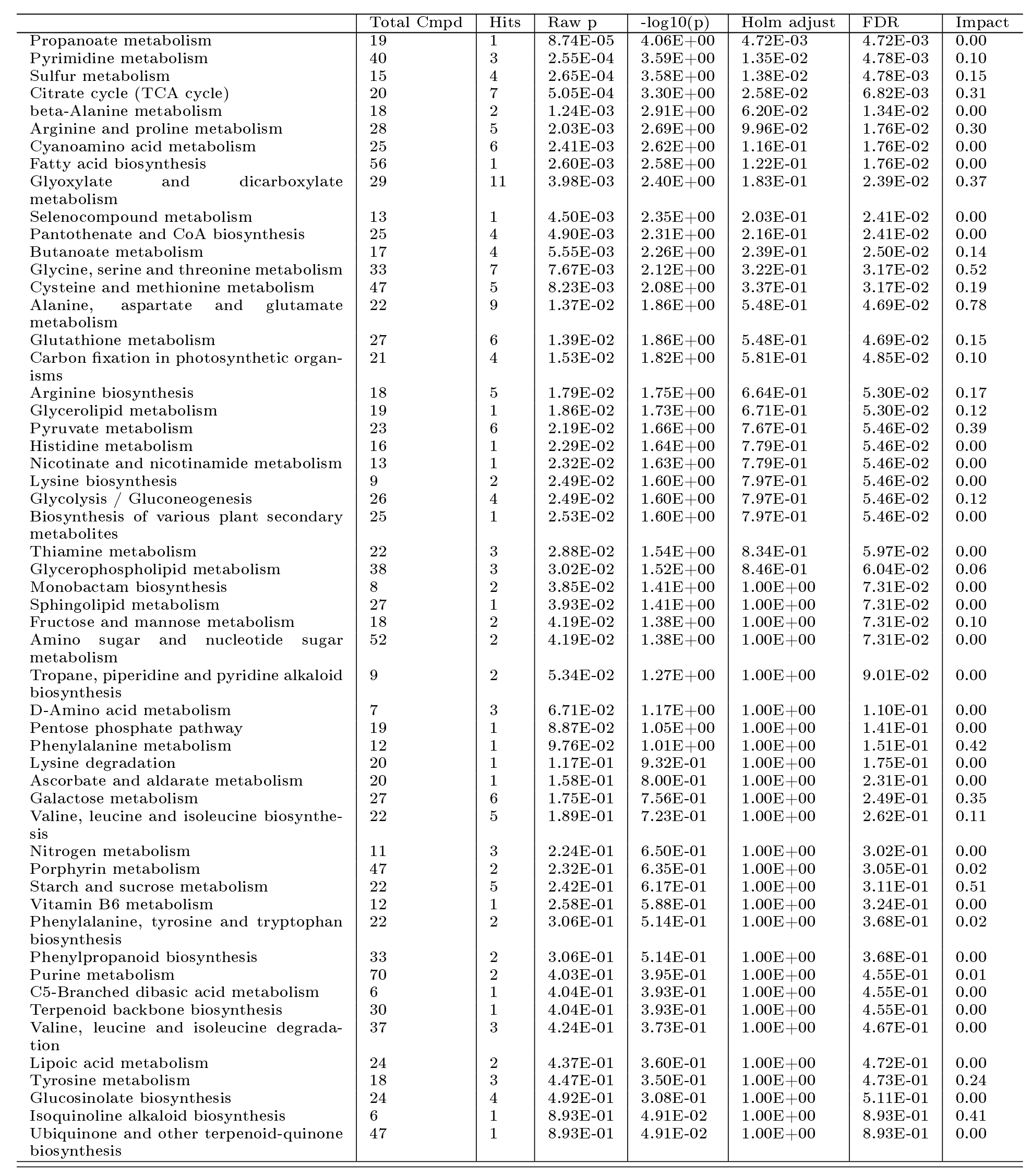


**Table S3.** The results of quantitative pathway analysis for Al-tolerant Demir-2000 roots treated with 30 µM AlCl_3_.6H_2_O. FDR indicates that *p*-values were adjusted using the false discovery rate. The impact of pathways was estimated by topology analysis.


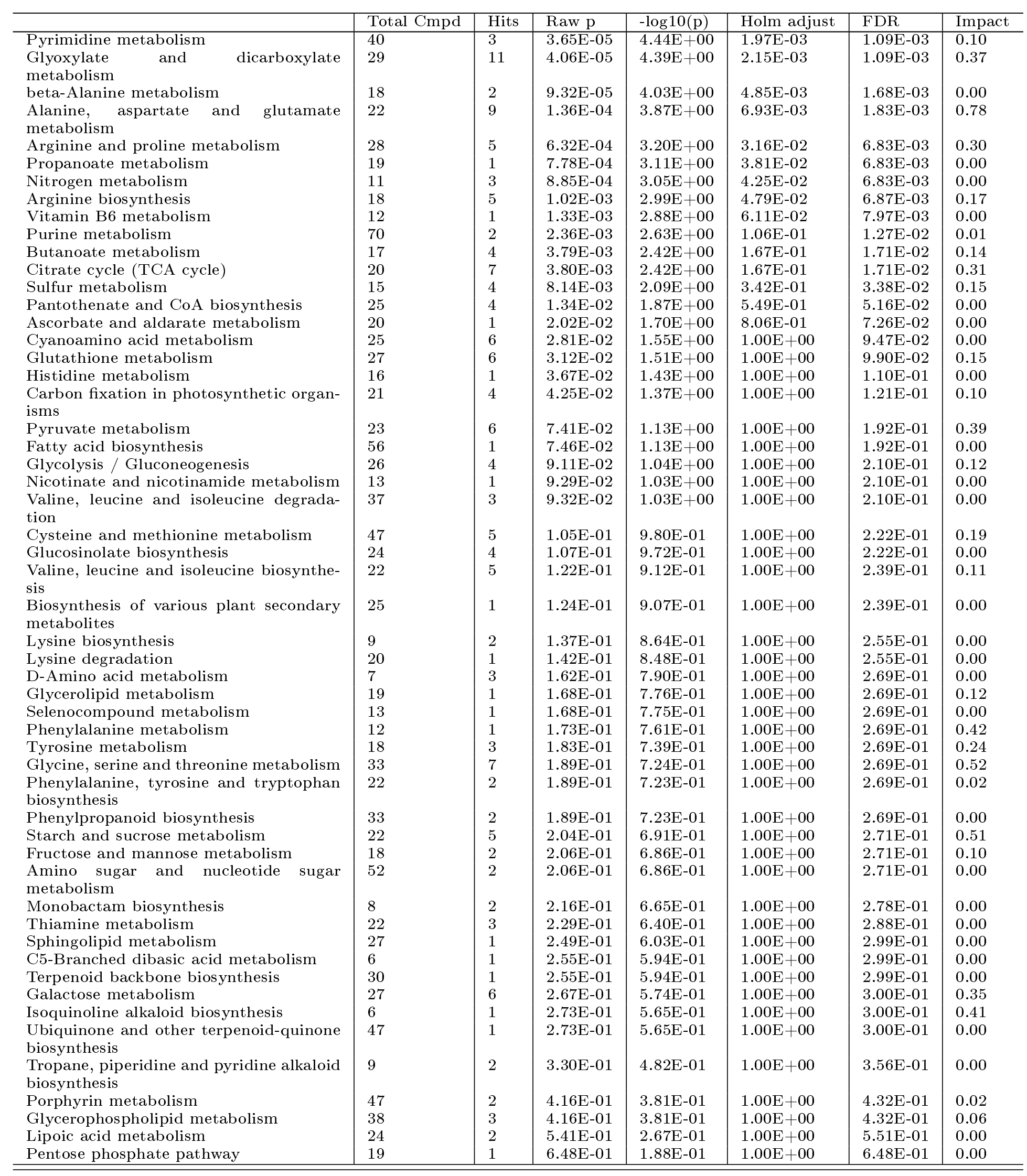


**Table S4.** The results of quantitative pathway analysis for Al-sensitive Golia-99 roots treated with 10 µM AlCl_3_.6H_2_O. FDR indicates that *p*-values were adjusted using the false discovery rate. The impact of pathways was estimated by topology analysis.


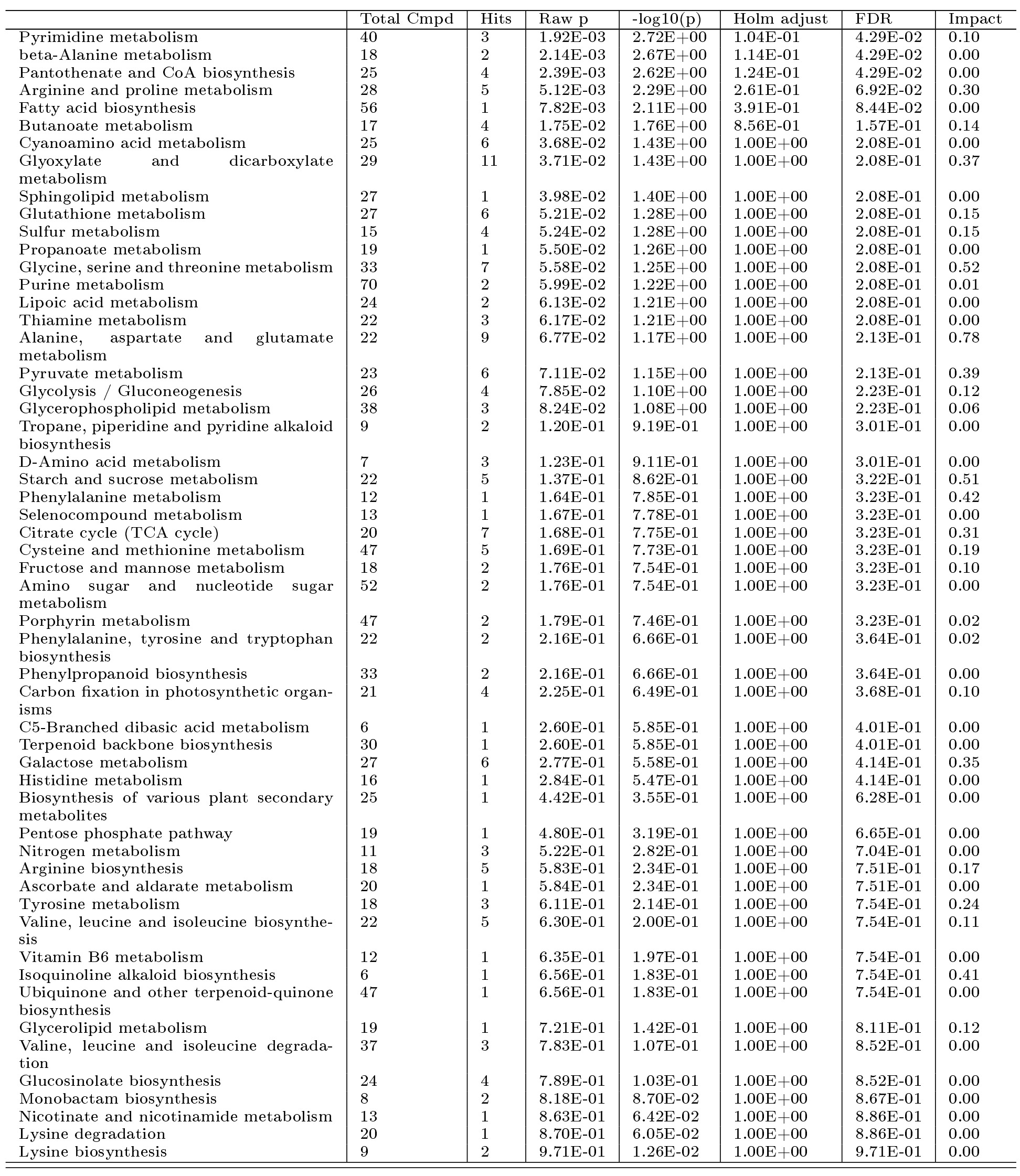


**Table S5.** The results of quantitative pathway analysis for Al-sensitive Golia-99 roots treated with 30 µM AlCl_3_.6H_2_O. FDR indicates that *p*-values were adjusted using the false discovery rate. The impact of pathways was estimated by topology analysis.


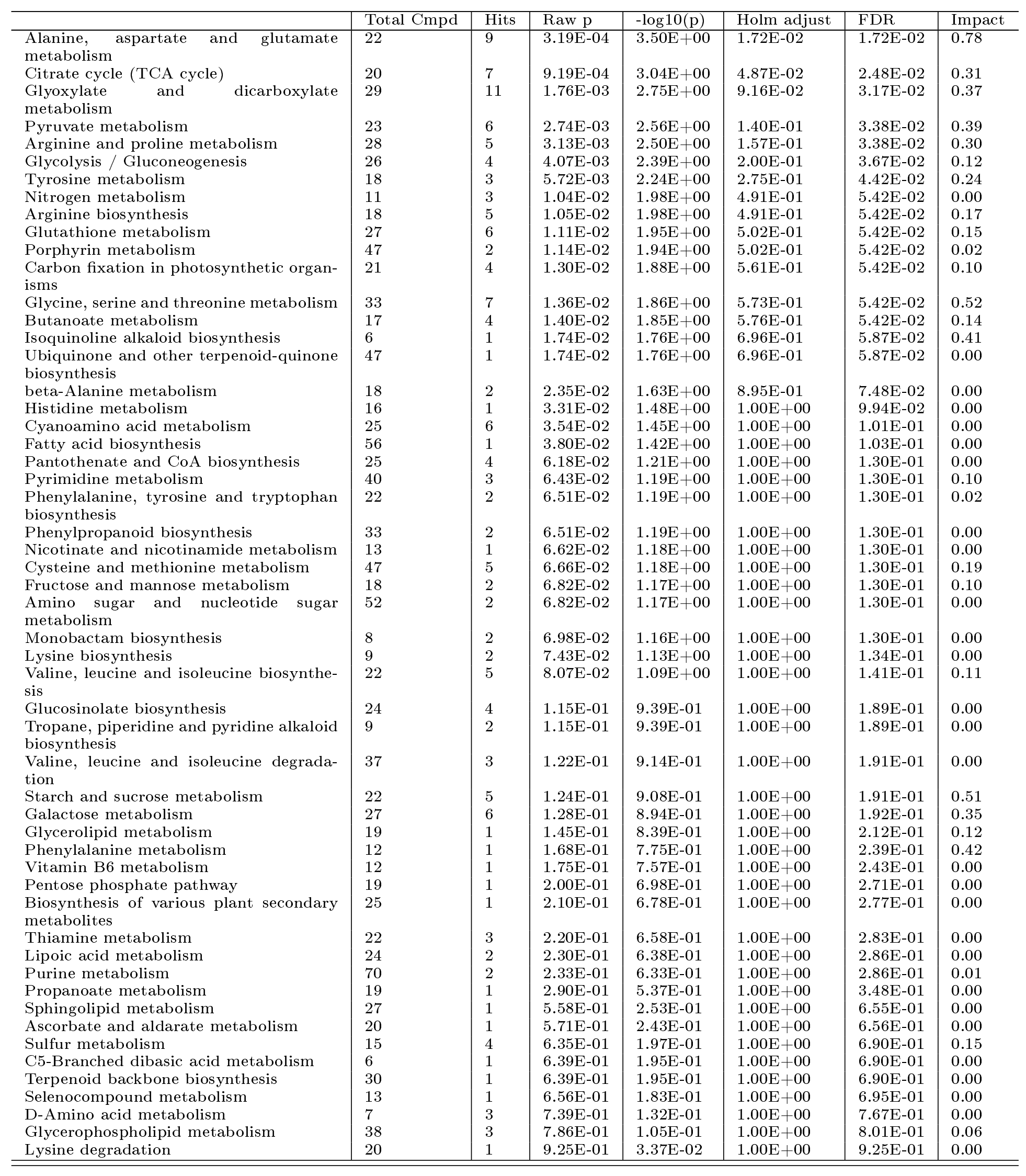

Supplement: Supplementary file 1 — Supplementary file1 (DOCX 1949 kb) [file 12298_2025_1622_MOESM1_ESM.docx]
